# Disentangling consequences of self-perceived weight from excess adiposity in metabolically healthy overweight/obesity

**DOI:** 10.1186/s12889-025-24254-2

**Published:** 2025-08-25

**Authors:** Bobby K. Cheon, Julia M. P. Bittner, Meegan R. Smith, Zhen Chen

**Affiliations:** 1 Social & Behavioral Sciences Branch, Division of Population Health Research, Eunice Kennedy Shriver National Institute of Child Health and Human Development, National Institutes of Health, Bethesda, MD USA; 2 Biostatistics and Bioinformatics Branch, Division of Population Health Research, Eunice Kennedy Shriver National Institute of Child Health and Human Development, National Institutes of Health, Bethesda, MD USA

**Keywords:** Weight perception, Metabolically healthy obesity, Metabolic health, Weight stigma, Longitudinal research

## Abstract

**Background:**

Greater self-perceived weight is detrimental to cardiometabolic health among people with overweight/obesity. However, it is impractical to test and unknown whether weight-related psychosocial factors, like self-perceived weight, independently affect cardiometabolic health apart from physiological dysregulation produced by excess adiposity among people with overweight/obesity. Metabolically healthy overweight/obesity (MHOv/Ob) involves overweight/obesity, but with the absence of metabolic dysfunction. The MHOv/Ob phenotype may be a promising model to examine unique contributions of self-perceived weight to long-term changes in metabolic health. We tested whether self-perceived weight independently contributes to declining metabolic health by comparing people with MHOv/Ob and metabolically healthy normal weight (MHNW). We hypothesized that: (1) those with MHOv/Ob are more likely to become metabolically unhealthy and gain BMI at follow-up (7–11 years later) compared to MHNW, and (2) these transitions in metabolic health and BMI among those with MHOv/Ob will be mediated by higher self-perceived weight.

**Methods:**

Using data from the National Longitudinal Study of Adolescent to Adult Health (Add Health), we classified metabolically healthy participants by weight status (normal weight, overweight, obesity) at Wave IV (ages 25–33). We tested whether longitudinal transitions from a metabolically healthy to unhealthy state (*n* = 788) and changes in BMI (*n* = 901) among MHOv/Ob (vs. MHNW) participants were mediated by higher self-perceived weight at Wave V (ages 33–43).

**Results:**

The MHOv/Ob (vs. MHNW) groups had greater odds of becoming metabolically unhealthy at Wave V (overweight odds ratio [OR]: 3.85 [1.87, 7.94], obesity OR: 9.12 [5.01, 16.61]). However, those with metabolically healthy obesity exhibited decreasing BMI (β: − 0.98 [− 1.75, − 020]). Although self-perceived weight was higher among the MHOv/Ob group, it did not mediate the relationship between Wave IV weight status and Wave V metabolic health (indirect effects—overweight risk difference (RD): 0.0046 [− 0.025, 0.033]; obesity RD: 0.0095 [− 0.050, 0.071]) or change in BMI (indirect effects—overweight β: − 0.30 [− 2.38, 1.75]; obesity β: − 0.51 [− 4.02, 2.84]).

**Conclusions:**

Self-perceived weight alone may not contribute to long-term metabolic dysfunction over-and-above physiological strain of excess adiposity among those living with overweight/obesity. However, future research on determinants of MHOv/Ob phenotypes should examine the role of other weight-related psychosocial factors such as weight stigma.

**Supplementary Information:**

The online version contains supplementary material available at 10.1186/s12889-025-24254-2.

## Introduction

While obesity is linked with poorer physical health [1], weight-related psychosocial factors, such as perceiving oneself as having more weight, may also contribute to future weight gain and health risks, independent of actual weight status [2–4]. Higher self-perceived weight does not necessary involve negative evaluations of oneself or weight stigma. However, higher self-perceived weight may influence weight gain and poorer health outcomes by increasing concerns about weight-based social rejection and internalization of weight stigma [5, 6]. Given that higher self-perceived weight may pose similar risks to cardiometabolic health as overweight/obesity [7, 8], it remains unclear whether these experiences contribute to long-term deterioration in physical health independent of the effects of overweight/obesity (excess adiposity) itself. The objective of this analysis is to examine whether differences in self-perceived weight may partially explain the long-term decline in metabolic health and increasing BMI among individuals with the metabolically healthy overweight/obesity phenotype (absence of metabolic dysfunction) compared to those with normal weight.

### Risks to cardiometabolic health associated with higher self-perceived weight

The perception of having overweight/obesity may impose significant psychosocial stressors that increase the risk of continued weight gain and physiological dysregulation. Although self-perceived weight is associated with actual BMI [7], self-perceived and actual weight are distinct given that the prevalence of misperceiving one’s weight status. Among a nationally-representative U.S. sample living with overweight or obesity, approximately 25% misperceived their weight status, and the relationship between weight misperception and weight loss behaviors varied based on sex and race [9]. Among adolescents, self-perceived overweight increased odds of suicidal ideation compared to the absence of perceived overweight, regardless of objective weight status [10]. Self-perceived overweight is proposed to heighten psychological distress from anticipated and self-directed weight stigma, which promotes excess energy intake and future gain of actual weight through increasing emotional eating, disrupting cognitive resources involved in regulating eating, or undermining self-efficacy to manage healthier diets [2, 6, 11–14]. For example, Robinson, Hunger and Daly (2015) observed across multiple longitudinal studies that self-perceived overweight predicts risk of future weight gain, potentially through stress-induced eating [3]. Thus, potential interventions that target self-perceived weight, such as reducing misperceptions of oneself as being overweight could be protective against stress-induced or dysregulated eating.

Self-perceived overweight may also contribute to maladaptive physiological and cardiometabolic responses. Experiencing weight stigma may trigger elevated cortisol responses [15, 16] and increased energy intake [12], both cross-sectionally and experimentally, especially among those who perceived themselves as overweight. Likewise, self-perceived overweight prospectively predicted worsening cardiometabolic health, a relationship that was mediated by weight gain [7]. Conversely, self-perceived normal-weight status may have a protective effect, predicting lower blood pressure in adulthood among adolescents with overweight/obesity [17]. Perceived weight discrimination, which may increase self-perceived overweight [18], has been associated with higher circulating levels of markers of systemic inflammation, such as C-reactive protein [19], and exacerbated relationships between high waist-to-hip ratio and glycemic control (measured by glycosylated hemoglobin; HbA1c) [20]. Notably, perceived weight discrimination may double the risk of high allostatic load, a summary measure of dysregulation across the nervous, cardiovascular, metabolic, and inflammatory systems, over a 10-year period [21]. Similarly, a 4-year longitudinal study observed that weight discrimination mediated the relationship between obesity and physiological dysregulation based on allostatic load indicators [22].

Although prior studies have proposed that greater self-perceived weight contributes to actual weight gain and worsening cardiometabolic health, a fundamental, yet unanswered question is whether this perception contributes to deteriorating cardiometabolic health independent of the physiological perturbations produced by excess adiposity of overweight/obesity itself. This question is impractical to answer directly since the negative psychological, physiological, and behavioral consequences of perceiving oneself as having excessive weight (e.g., increased psychological distress, multi-system allostatic load, increased food intake) are intractably confounded with physiological dysregulation caused by overweight/obesity.

One way to address this question is by statistically controlling for participants’ baseline cardiometabolic health profiles when examining the effect of weight-related psychosocial factors on long-term cardiometabolic health outcomes. However, this is rarely done beyond controlling for overall self-rated health or self-reported health conditions, since direct measures of baseline cardiometabolic health may not be available in many longitudinal cohorts. In one study, self-perceived overweight longitudinally predicted poorer cardiometabolic functioning while controlling for baseline measures of cardiometabolic functioning indirectly estimated from data available in other cohorts given the lack of baseline measurements in the target cohort [7]. Another longitudinal study showed that higher perceived weight discrimination was associated with worsened cardiometabolic health, even when controlling for baseline physiological dysregulation and BMI [22]. Another finding revealed a similar relationship between perceived weight discrimination and worsened glycemic control among a non-diabetic sample [20]. However, these studies examined the effects of encountering explicit weight-based discrimination rather than self-perceived weight. Experimental approaches, such as random assignment to wearing a prosthetic that creates the appearance of obesity (vs. control), have also sought to disentangle the effects of the experience and self-perception of having overweight/obesity from the biological strain of excess adiposity on outcomes such as negative affect, consumption of unhealthy snack foods, and stress responses [23]. Yet these acute experiments are not able to capture long-term and cumulative effects of perceiving oneself as overweight/obese on health outcomes.

A design that directly compares people who have overweight/obesity yet are metabolically healthy with those who have normal weight and are metabolically healthy would provide a more accurate test of the independent effect of the psychosocial experience of having overweight/obesity on future cardiometabolic health outcomes. Although it is impractical to randomly assign people to a lived experience of perceiving oneself as having excess weight while being metabolically healthy, this aim could be achieved through the study of the metabolically healthy obesity phenotype as a potential model for testing the independent effects of weight-related perceptions on health.

### The metabolically healthy obesity phenotype

Obesity and dysmetabolic status are typically associated, yet some individuals with obesity are metabolically healthy [24]. Referred to as the metabolically healthy obesity (MHO) phenotype, these individuals have BMIs that exceed the threshold of obesity (BMI ≥ 30 kg/m^2^) but have insufficient markers of metabolic dysfunction (e.g., metabolic syndrome) [25–27]. As such, comparisons of MHO with metabolically healthy and normal weight (MHNW; 18.5 ≤ BMI < 25 kg/m^2^) individuals may provide an opportunity to determine the contributions of self-perceived overweight to future metabolic abnormalities, independent of current metabolic dysfunction that may be comorbid with overweight/obesity.

Prior research has suggested that those with the MHO phenotype are indeed healthier and at lower risk of cardiometabolic disease than metabolically unhealthy individuals of any weight. Compared to people with metabolically unhealthy obesity (MUO), MHO individuals had a higher fitness level and a 38% lower risk of all-cause mortality [28]. Moreover, cardiovascular disease (CVD) risk for MHO individuals is lower compared to metabolically unhealthy normal weight (MUNW) and risk is not higher compared to MHNW, indicating that MHO may be a benign condition [29–31].

However, other studies have demonstrated that regardless of metabolic status, obesity is associated with non-cardiovascular death, cancer, myocardial infarction, and ischemic heart disease [32, 33]. Compared to MHNW, MHO may be a higher risk state for CVD and associated events, type 2 diabetes, and all-cause mortality [27, 34–38]. Similarly, there are findings suggesting MHO status may not be stable in the long-term, such that people living with MHO are at risk of eventually transitioning into metabolically unhealthy status. MHO participants have exhibited significantly lower high-density lipoprotein cholesterol levels and higher blood pressure, total cholesterol, and glucose levels than MHNW participants indicating that MHO may be part of the process of becoming metabolically unhealthy [27]. This transition from a metabolically healthy to unhealthy status among individuals with overweight and obesity has been described as a long-term process that could span approximately decade or more [32, 38].

These controversies and inconsistencies about the stability and health risks of the MHO phenotype may be explained by stressors associated with higher self-perceived weight, since these influences increase risk for future gain of actual weight, stress-induced eating, and cardiometabolic abnormalities [3, 5, 7]. Thus, the experience of self-perceived weight may be an unexplored yet important determinant of the health trajectories and stability of the MHO phenotype. Despite the promise of self-perceived weight as a potential explanation or determinant for inconsistencies in the cardiometabolic health trajectories of MHO, no studies to our knowledge have tested whether progression of MHO into continued weight gain or deteriorating cardiometabolic health is associated with self-perceived weight or other weight-related psychosocial factors, such as weight stigma.

### Present study

The current study compared the MHO phenotype with MHNW to test whether greater self-perceived weight even in the absence of cardiometabolic dysregulation contributes to long-term (approximately 10 years) decline in cardiometabolic health and increased BMI of participants with MHO. Our analyses included both metabolically healthy overweight and obesity (MHOv/Ob), since those who have overweight or obesity are both more likely to perceive themselves as overweight compared to those who are normal weight [18]. We hypothesized (pre-registered) that participants with MHOv/Ob would exhibit worse cardiometabolic health (Hypothesis 1 A) and increased BMI (Hypothesis 1B) at follow-up compared to participants with MHNW. We also hypothesized that worsening metabolic health (Hypothesis 2 A) and increased BMI (Hypothesis 2B) for MHOv/Ob participants would be mediated by greater self-perceived weight.

## Methods

The hypotheses, design, and analyses for this project were pre-registered^1^ on Open Science Framework (osf.io/b9e4z).

### Study sample

Data for this analysis came from the National Longitudinal Study of Adolescent to Adult Health (Add Health) [39]. Add Health recruited a nationally representative sample of adolescents in grades 7 − 12 in 1994 − 1995 and has longitudinally followed participants through 2018 with the goal of understanding the health and social contexts of individuals as they transition from adolescence into adulthood. Details of the Add Health sampling design and survey materials have been published elsewhere [40]. Briefly, stratified sampling was used to randomly select high schools from the Quality Education Database. Among participating schools, a random sample of students were selected to participate in In-Home surveys at baseline (Wave I, 1994 − 1995) and longitudinally thereafter. Ethical approval was not required for this project as analyses were conducted on publicly available, de-identified Add Health data.

From the 3,713 individuals who participated in Waves I (aged 11–21), IV (2008–2009, aged 25–33) and V (2016–2018, aged 33–43) data collection, participants were excluded if they were pregnant during Wave IV or V data collection (*n* = 195), were missing a blood sample at Wave IV (*n* = 226), did not participate in the Wave V home exam [anthropometrics, blood draw] (*n* = 1,688), or were underweight at Wave IV (*n* = 49). Analyses of metabolic health (Hypotheses 1 A, 2 A) additionally excluded individuals who had an unsuccessful blood draw at Wave V (*n* = 118), were not metabolically healthy at Wave IV (*n* = 588), missing Wave IV BMI (*n* = 6), missing metabolic health status at Wave V (*n* = 52), or were missing covariates (*n* = 3). Analyses of BMI (Hypotheses1B, 2B) additionally excluded those who were not metabolically healthy at Wave IV (*n* = 632), were missing Wave IV BMI (*n* = 7), missing BMI at Wave V (*n* = 11), or missing covariates (*n* = 4). After exclusions, the analytic sample size for Hypothesis A analyses was 788 and the analytic sample size for Hypothesis B analyses was 901.

### Measures

Height and weight (to compute BMI) were measured by trained Add Health fieldworkers. Weight was measured to the nearest 0.1 kg using a Health-O-Meter 844KL digital scale and height was measured to the nearest 0.5 cm using a carpenter scale and steel tape measure.

Wave IV weight status, the exposure, was determined using the conventional body mass index (BMI) cut-offs of 18.5 kg/m^2^ *≤* BMI < 25 kg/m^2^ for normal weight, 25 kg/m^2^ *≤* BMI < 30 kg/m^2^ for overweight, and BMI ≥ 30 kg/m^2^ for obesity. Change in BMI between Waves IV and V (Hypothesis B outcome) was calculated as the continuous difference between Waves IV and V BMI.

Metabolic health status was a binary variable (healthy or unhealthy). Participants were considered metabolically unhealthy if they met two or more of the following Metabolic Syndrome criteria outlined by the International Diabetes Federation [41]: high triglyceride levels (*≥* 150 mg/dL), low high-density lipoprotein (HDL) cholesterol levels (males: <40 mg/dL, females: <50 mg/dL or antihyperlipidemic medication use), high blood pressure (systolic blood pressure ≥ 130 mmHG or diastolic blood pressure ≥ 85 mmHG), or high blood glucose (fasting plasma glucose *≥* 100 mg/dL or anti-diabetic medication). Add Health did not report fasting glucose and only reported deciles of triglycerides and HDL; therefore, high-risk cut-offs for these indicators were determined using glycated hemoglobin (HbA1c ≥ 5.7%) instead of fasting plasma glucose and deciles, rather than concentrations, of triglycerides (males: in top three deciles, females: in top two deciles) and HDL (males: in bottom two deciles, females: in bottom three deciles), as has been done in previous Add Health studies of Metabolic Syndrome [42, 43]. Worsening metabolic health (Hypothesis A outcome) was defined as developing Metabolic Syndrome by Wave V (vs. remaining metabolically healthy at Wave V). Participants were therefore considered MHOv/Ob at Wave IV if their BMI placed them in the overweight or obese categories and they were categorized as metabolically healthy.

Self-perceived weight (Wave IV), the mediator, was determined via participants’ response to the question “How do you think of yourself in terms of weight?” with response options ranging from 1 (very underweight) to 5 (very overweight) and operationalized continuously.

Covariates included age (Wave IV; continuous), sex (male, female), race/ethnicity (non-Hispanic white, non-Hispanic Black, Hispanic, other race(s)), educational attainment (Wave IV; some high school or less, graduated high school, vocational/technical school/some college, completed college, at least some post graduate schooling), and smoking status (Wave IV; ever, never smoked in last 30 days).

### Statistical analyses

Descriptive statistics were separately calculated among those eligible for Hypothesis A and B analyses. Adjusted logistic regressions were used to examine relationships between Wave IV weight status and changes in metabolic health status between Waves IV and V (Hypothesis 1 A). Average predicted probabilities of having worsening metabolic health status (while controlling for covariates) and differences in the risk of worsening metabolic health status between individuals with normal weight and those with overweight or obesity were estimated from the logistic regressions. Adjusted linear regressions were used to examine relationships between Wave IV weight status and changes in BMI between Waves IV and V (Hypothesis 1B). Linear regressions confirmed relationships between Wave IV weight status and self-perceived weight (mediation analysis assumption) and logistic and linear regressions confirmed relationships between self-perceived weight and the outcomes (mediation analysis assumption). Mediation via self-perceived weight (Hypotheses 2 A and 2B) was tested using the method described by Imai and colleagues [44]. Estimates of the total effect (full relationship between Wave IV weight status and the outcomes), direct effect (the relationship between Wave IV weight status and outcomes not attributable to self-perceived weight), and indirect effect (the relationship between Wave IV weight status and outcomes operating through self-perceived weight) were obtained. All analyses adjusted for the covariates and accounted for the complex sampling design employed by Add Health. Analyses were conducted in Stata version 16.1 (College Station, TX, USA) and using the *mediation* package (with 10,000 quasi-Bayesian Monte Carlo simulations) [45] in R 4.1.2 (Vienna, Austria).

## Results

Sociodemographic characteristics were similar in both samples: participants’ average age at Wave IV was 28 years, nearly two-thirds of participants were female (Hypothesis A sample: 65%, Hypothesis B sample: 64%) and three-quarters were non-Hispanic White (Hypothesis A: 77%, Hypothesis B 75%; Table 1). Average self-perceived weight at Wave IV was 3.59 in both samples. Distributions of Wave IV weight category were similar across the two samples, with approximately 41% of participants having normal weight, 28% having overweight, and 30% having obesity. An average of 8.95 years passed between participation in Waves IV and V (range: 7.47–11.20 years).

**Table 1:** Descriptive statistics of metabolic health, weight status, self-perceived weight, and covariates among participants in the add health study eligible for the hypothesis 1 A/2A and the hypothesis 1B/2B analyses

Covariates	Sample for Hypotheses 1 A & 2 A (*n* = 788)	Sample for Hypotheses 1B & 2B (*n* = 901)
	Percent (*n*)^a^	Percent (*n*)^a^
Age (Wave IV; mean [SD])	28.34 (1.85)	28.35 (1.84)
Sex		
Female	64.60 (550)	64.31 (632)
Male	35.40 (238)	35.69 (269)
Race/ethnicity		
Non-Hispanic White	76.81 (574)	75.31 (639)
Non-Hispanic Black	11.77 (131)	12.73 (164)
Hispanic	8.99 (61)	8.68 (69)
Other	2.43 (22)	3.28 (29)
Education (Wave IV)		
Some high school	6.21 (36)	6.59 (43)
Graduated high school	10.93 (75)	11.11 (88)
Some college or technical training	43.73 (319)	42.84 (360)
Graduated college	23.62 (200)	23.96 (229)
At least some graduate school	15.52 (158)	15.50 (181)
Smoking (Wave IV)		
Smoked in last 30 days	35.68 (228)	36.49 (270)
Did not smoke in the last 30 days	64.32 (560)	63.51 (631)

Mediator	Mean (SD)	Mean (SD)
Self-perceived weight (Wave IV) Range: 1–5	3.59 (0.81)	3.59 (0.82)

	Percent (n)^a^	Percent (n)^a^
*Metabolic health*		
Metabolic health at Wave V		
Metabolically healthy	78.55 (641)	---
Metabolically unhealthy	21.45 (147)	---
*Weight status*		
Weight Category at Wave IV		
Normal	41.13 (330)	41.48 (374)
Overweight	28.66 (229)	27.91 (257)
Obesity	30.21 (229)	30.61 (270)
Change in BMI from Wave IV to Wave V (mean [SD])	–	1.83 (4.07)

*BMI body mass index; *SD* standard deviation

^a^Percentages weighted using Add Health sampling weights. Cell sizes are unweighted counts

### Hypotheses 1 A and 2 A: worsening metabolic health

Most participants (79%) remained metabolically healthy between Waves IV and V. The average predicted probabilities of having worsening metabolic health were 0.075 (95% CI: 0.041, 0.11) for individuals with normal weight at Wave IV, 0.23 (95% CI: 0.15, 0.30) for those with overweight, and 0.40 (95% CI: 0.33, 0.47) for those with obesity. Wave IV weight status was associated with worsening metabolic health by Wave V; compared to those in the normal weight category at Wave IV, the average risk of worsening metabolic health between Waves IV and V was 15% points (95% CI: 0.066, 0.24) and 32% points (95% CI: 0.24, 0.40) higher for participants with overweight and obesity at Wave IV, respectively (Hypothesis 1 A; Table 2). Wave IV weight status was also associated with self-perceived weight, such that those with overweight reported an average 0.69-unit (95% CI: 0.59, 0.79) higher self-perceived weight value and those with obesity reported an average 1.17-unit (95% CI: 1.05, 1.29) higher self-reported weight value, compared to participants with normal weight (mediation ‘a’ path). However, self-perceived weight was not associated with metabolic health status at Wave V when adjusting for Wave IV weight status (risk difference: 0.0082, 95% CI: -0.044, 0.060; mediation ‘b’ path). Mediation analysis demonstrated no indirect effect of Wave IV weight status on Wave V metabolic health through self-perceived weight (overweight risk difference: 0.0046, 95% CI: -0.025, 0.033; obesity risk difference: 0.0095, 95% CI: -0.050, 0.071; Hypothesis 2 A; Table 3).

**Table 2:** Unadjusted and adjusted results of analyses examining relationships between wave IV weight category with metabolic health (Hypothesis 1 A) and change in BMI (Hypothesis 1B), among eligible participants in the add health study

Hypothesis 1 A: Association between wave IV weight category and wave V metabolic health (*n* = 788)		
Wave IV weight category	Unadjusted risk difference (95% CI)	Adjusted risk difference (95% CI)
Normal weight	Ref	Ref
Overweight	**0.17 (0.084**,** 0.25)**	**0.15 (0.066**,** 0.24)**
Obesity	**0.30 (0.22**,** 0.38)**	**0.32 (0.24**,** 0.40)**

Hypothesis 1B: Association Between Wave IV Weight Category and change in BMI between Wave IV and Wave V (n = 901)		
Wave IV Weight Category	Unadjusted β (95% CI)	Adjusted β (95% CI)
Normal weight	Ref	Ref
Overweight	0.48 (− 0.19, 1.15)	0.45 (− 0.18, 1.08)
Obesity	**− 0.88 (− 1.64**,**− 0.11)**	**− 0.98 (− 1.75**,**− 0.20)**

*BMI* body mass index, *CI* confidence interval

Bold indicates statistical significance at the α = 0.05 level

Logistic regression models were used to examine changes in metabolic health and linear models were used to examine changes in BMI. Models were adjusted for Wave IV age, race, sex, Wave IV educational attainment, and Wave IV smoking status

**Table 3:** Results of analyses examining mediation of the relationships of wave IV weight category with metabolic health (Hypothesis 2 A) and change in BMI (Hypothesis 2B) via self-perceived weight, among eligible participants in the add health study

Hypothesis 2 A: Association Between Wave IV Weight Category and Wave V Metabolic Health, Mediated by Self-Perceived Weight (*n* = 788)			
Wave IV Weight Category	Direct Effects Risk Difference (95% CI)	Indirect Effects Risk Difference (95% CI)	Total Effects Risk Difference (95% CI)
Normal weight	Ref	Ref	Ref
Overweight	**0.15 (0.060**,** 0.24)**	0.0046 (− 0.025, 0.033)	**0.15 (0.071**,** 0.24)**
Obesity	**0.31 (0.20**,** 0.41)**	0.0095 (− 0.050, 0.071)	**0.32 (0.24**,** 0.39)**

Hypothesis 2B: Association Between Wave IV Weight Category and change in BMI between Wave IV and Wave V, Mediated by Self-Perceived Weight (n = 901)			
Wave IV Weight Category	Direct Effects β (95% CI)	Indirect Effects β (95% CI)	Total Effects β (95% CI)
Normal weight	Ref	Ref	Ref
Overweight	0.76 (− 0.010, 1.51)	− 0.30 (− 0.74, 0.14)	0.45 (− 0.18, 1.07)
Obesity	− 0.48 (− 1.54, 0.60)	− 0.51 (− 1.26, 0.24)	− 0.98 (− 1.74, − 0.21)

*BMI* body mass index, *CI* confidence interval

Bold indicates statistical significance at the α = 0.05 level

The R *mediation* package was used to estimate direct, indirect, and total effects. Models were adjusted for Wave IV age, race, sex, Wave IV educational attainment, and Wave IV smoking status

### Hypotheses 1B and 2B: change in BMI

Most participants’ (73%) BMI increased between Waves IV and V, with a mean change in BMI of 1.83 kg/m^2^ (range: -16.3 kg/m^2^ to 17.7 kg/m^2^, Table 1). Wave IV weight status was associated with BMI change for participants with obesity, but not those with overweight; compared to participants with normal weight, those with obesity had a 0.98-unit (95% CI: -1.75, -0.20) decrease in BMI but those with overweight did not have a significantly different BMI change compared to those with normal weight (β: 0.45, 95% CI: -0.18, 1.08; total (Hypothesis 1B; Table 2). As before, Wave IV overweight and obesity were both associated with higher self-perceived weight (overweight β: 0.71, 95% CI: 0.62, 0.81; obesity β: 1.19, 95% CI: 1.08, 1.31) but self-perceived weight was not associated with change in BMI when adjusting for Wave IV weight status (β: -0.43, 95% CI: -1.05, 0.20). Mediation analyses demonstrated no indirect effects (overweight β: -0.30, 95% CI: -0.74, 0.14; obesity β: -0.51, 95% CI: -1.26, 0.24) of Wave IV weight status on change in BMI between Waves IV and V (Hypothesis 2B; Table 3).

## Discussion

Although higher self-perceived weight and excess adiposity pose overlapping risks to metabolic health, there has been limited research to directly dissociate their independent risks to deteriorating metabolic health. This study examined the MHOv/Ob phenotypes as a model to test whether individuals who perceived themselves as having excess weight, yet are metabolically healthy, are at increased odds of worsening metabolic health and increased BMI 7–11 years later compared to lean and metabolically healthy counterparts.

Similar to prior studies on self-perceived weight [18], both the groups with overweight and obesity perceived themselves to have more weight than the normal weight group among our sample of metabolically healthy participants. Furthermore, the odds of having worsened metabolic health at follow-up (Wave V) were greater for both the overweight and obesity groups compared to the normal weight group (Hypothesis 1 A supported). This supports prior studies suggesting that the MHO phenotype may not be entirely benign and is associated with deteriorating metabolic health and greater risk of disease over time compared to normal weight [27, 32–37]. Unlike prior research, we observed that participants with obesity at Wave IV had decreasing BMIs compared to the participants with normal weight at follow-up during Wave V (Hypothesis 1B and 2B unsupported). Compared to participants who could maintain normal weight, those living with obesity may have been more likely to engage in treatments or lifestyle changes to reduce their weight.

Despite the MHOv/Ob groups reporting higher self-perceived weight and exhibiting worsening metabolic health than the normal weight group, self-perceived weight did not mediate the relationship between weight status at Wave IV and progression to a metabolically unhealthy state at Wave V (Hypothesis 2 A unsupported). Supplemental analyses that compared mean self-perceived weight among participants with MHOv/Ob who remained metabolically healthy versus became metabolically unhealthy at Wave V also revealed no differences in self-perceived weight between these groups (see Supporting Information; Table S1). One interpretation of this finding is that among people with overweight/obesity, self-perceived weight may not independently contribute to long-term decline in metabolic health over-and-above physiological dysregulation from excess adiposity. Consistent with this, our analyses also showed that higher self-perceived weight was not associated with either change in BMI or worsening metabolic health when controlling for baseline weight status. Perhaps exposure to more salient and stressful aspects of weight stigma, such as discrimination and mistreatment based on one’s weight, may independently contribute to deterioration of metabolic health among those with MHOv/Ob. However, this finding should not trivialize the well-documented pernicious effects of higher self-perceived overweight on health. Given that the effects of weight stigma on stress responses and increased food intake may be stronger among people who already perceive themselves as overweight [12, 16], future research examining the effects of self-perceived weight on health outcomes in the MHOv/Ob phenotype should also account for measures of exposure to weight stigma, such as perceived weight-based discrimination or internalized stigma. Furthermore, these findings are specific to MHO, which is a relatively infrequent subtype of overweight/obesity, affecting 10–15% of population-based samples [46–48]. Thus, self-perceived weight may still be a profound social determinant of long-term cardiometabolic health for most persons with overweight/obesity.

These findings contribute novel insights into the long-term health consequences of perceiving oneself as having greater weight. Although higher self-perceived weight is associated with increased weight gain and strain to multiple physiological systems [3, 4, 17] prior research has not directly tested these relationships among people with MHOv/Ob. Our findings showed that MHOv/Ob imposes greater long-term odds of progressing to a metabolically unhealthy status compared to MHNW, yet this change was not mediated by higher self-perceived weight experienced by the MHOv/Ob groups. This study also offers novel insights into controversies surrounding the long-term stability and harmlessness of the MHO phenotype. Prior research on MHO samples has not examined the role of weight-related psychosocial factors, such as self-perceived overweight, as a determinant of long-term health trajectories. Based on a large cohort that has not yet been examined in prior studies of the MHO phenotype, our findings provide a new source of support for the perspective that MHOv/Ob may represent a transitional phase in which overweight/obesity gradually progresses into metabolic disorder [32]. Although prior studies show that weight-related stressors, including those associated with self-perceived weight, predict declining cardiometabolic health [21, 22], our study demonstrates that perceiving oneself as having overweight may not be a determinant of whether people with MHOv/Ob maintain their metabolic health long-term.

Strengths of the study include a longitudinal sample from across the United States to examine changes in metabolic health and BMI across approximately 10 years. BMI was also assessed using measurements of height and weight rather than self-report. However, a limitation of the study is that our findings are specific to a sample of predominantly White American adults relatively early in adulthood (ages 24–32 to ages 33–43). The relationship between BMI and susceptibility to metabolic abnormalities can vary based on both race and age [49, 50]. Additionally, results cannot be interpreted causally due to the presence of unmeasured confounding inherent in all observational studies. Further, about half (56%) of participants who completed the Wave V survey did not complete the home exam (anthropometrics, blood draw). This could potentially bias the results if individuals who feel they have a high bodyweight are less likely to participate in the home exam. However, self-perceived weight was similar between those who did (mean = 3.61) and did not (mean = 3.65) complete the home exam, suggesting those with higher bodyweight may not be less likely to participate in the home exam. Moreover, the exposure and the mediator were measured during the same wave of data collection, which prevents the establishment of temporality between these two variables. We were also limited to examining self-perceived weight as the only weight-related psychosocial factor. While self-perceived weight is associated with stigma-related concerns [5, 6], it does not necessarily reflect the presence of self-stigma or exposure to weight-based mistreatment. Add Health had a measure of reasons for perceived mistreatment by others, which included one’s weight as a response option. However, there was a limited number of participants that selected this option. Another limitation of this study is the classification of participants as having excess adiposity (overweight or obesity status) based on BMI, rather than waist circumference or a direct measure of adiposity, such as dual-energy X-ray absorptiometry (DXA). However, given the relative infrequency of the MHO phenotype in the population, the current study required a large cohort. Directly assessing adiposity on such a large cohort may be less practically feasible, but large cohort studies with these measures should also consider including measures related to self-perceived weight or weight stigma. In sum, this approach of investigating the MHO phenotype to isolate and test the unique contributions of self-perceived overweight to health should be replicated in samples of other ages (e.g., adolescents), racial/ethnic backgrounds, and with other measures of weight-related psychosocial factors, such as weight stigma.

In conclusion, we demonstrate that while individuals with MHOv/Ob are more likely to become metabolically unhealthy than individuals with MHNW over 7–11 years, this difference was not attributable to higher levels of self-perceived weight among those with MHOv/Ob. However, given that physiological processes dysregulated by the excess adiposity of obesity such as maintenance of positive energy balance, inflammation, dyslipidemia, and hypertension are absent in MHO while also being consequences of psychosocial stressors, this study reveals the promise of integrating the study of the MHO phenotype with health consequences of weight-related psychosocial factors. MHO may not only be a valuable model for understanding how excess adiposity generates cardiometabolic complications through physiological mechanisms [24], but also through psychosocial ones. Future studies should test the role of weight stigma as a determinant of the progression of MHOv/Ob to a metabolically unhealthy state.

## Supplementary Information

Below is the link to the electronic supplementary material.

Supplementary Material 1

## Data Availability

Data from this study is publicly available from the Add Health Study website (https://addhealth.cpc.unc.edu/data/).

## Footnotes

1 Analyses presented in this paper slightly deviated from the original preregistration to accommodate feedback received on an earlier version of this manuscript. Poor/disrespectful treatment by others and inflammation were removed as potential psychosocial risk factors related to weight gain and the progression from metabolically healthy to metabolically unhealthy, as these factors are not directly weight related. Change in weight from Wave IV to Wave V was made continuous to allow for a more sensitive examination of relationships between baseline weight category, self-perceived weight, and weight change. Waist-to-height ratio was removed from the operationalization of baseline weight category to utilize a more standard operationalization of weight category. A formal test of mediation was used rather than the preregistered comparison of mean levels of self-perceived weight by baseline weight categories to increase rigor and interpretability of mediation analysis results.
