# Supplementary material for: Disentangling consequences of self-perceived weight from excess adiposity in metabolically healthy overweight/obesity
Source: BMC Public Health. 2025 Aug 25;25:2907. doi: 10.1186/s12889-025-24254-2 (PMC12376466; doi:10.1186/s12889-025-24254-2)
Supplement: Supplementary file 1 — Supplementary Material 1 [file 12889_2025_24254_MOESM1_ESM.docx]

**Supporting Information For:**

**Disentangling consequences of self-perceived weight from excess adiposity in metabolically healthy overweight/obesity**

**Supplemental Analyses**

*Comparisons of self-perceived weight between participants with metabolically healthy overweight/obesity that remained metabolically healthy and became metabolically unhealthy*

Supplemental analyses were conducted to determine whether there were differences in self-perceived weight reported at Wave IV between metabolically healthy overweight or obese participants who became metabolically unhealthy or remained metabolically healthy at Wave V. Thus, this analysis sought to test whether participants who progressed from metabolically healthy overweight/obesity (MHOv/Ob) to a metabolically unhealthy status in Wave V experienced greater self-perceived weight compared to those who those with MHOv/Ob who remained metabolically healthy.

In this analysis, mean self-perceived weight was compared between four groups of participants: 1) metabolically healthy with overweight at Wave IV and metabolically healthy at Wave V, 2) metabolically healthy with obesity at Wave IV and metabolically healthy at Wave V, 3) metabolically healthy with overweight at Wave IV and metabolically *un*healthy at Wave V, and 4) metabolically healthy with obesity at Wave IV and metabolically *un*healthy at Wave V. Analyses were conducted using analysis of covariance (ANCOVA) comparing self-perceived weight between these four groups while controlling for the same covariates as our primary analyses: age (Wave IV; continuous), sex (male, female), race/ethnicity (non-Hispanic white, non-Hispanic Black, Hispanic, other race(s)), educational attainment (Wave IV; some high school or less, graduated high school, vocational/technical school/some college, completed college, at least some post graduate schooling), and smoking status (Wave IV; ever, never smoked in last 30 days).

Among participants with metabolically healthy overweight and obesity at Wave IV, mean self-perceived weight was highest among those who were metabolically healthy with obesity at Wave IV and remained metabolically healthy at Wave V (“Group 2”; mean self-reported health: 4.03; Table S1) and those who were metabolically healthy with obesity at Wave IV and became metabolically unhealthy by Wave V (“Group 4”; 4.03), followed by those who were metabolically healthy with overweight at Wave IV and remained metabolically healthy at Wave V (“Group 1”; 3.56), and those who were metabolically healthy with overweight at Wave IV and became metabolically unhealthy by Wave IV (“Group 3”; 3.52). ANCOVA analyses indicated there were no statistically significant differences in mean self-perceived weight between those with overweight who remained metabolically healthy vs those who became metabolically unhealthy by Wave V (contrast: 0.042, 95% CI: -0.13, 0.22) nor were there statistically significant differences in mean self-perceived weight between those with obesity who remained metabolically healthy vs those who became metabolically unhealthy by Wave V (contrast: -0.00080, 95% CI: -0.17,0.17).

Together, results of these supplemental analyses suggest that among metabolically health participants at Wave IV, those with obesity overall experienced higher self-perceived weight than those with overweight. However, there was no indication that those who became metabolically unhealthy at Wave V experienced higher self-perceived weight than those who continued to remain metabolically healthy. This is demonstrated by no differences in self-perceived weight between the overweight group that remained metabolically healthy vs. became metabolically unhealthy (Group 1 – Group 3 on Table S1) or between the obesity group that remained metabolically healthy vs. became unhealthy (Group 2 – Group 4 on Table S1). These supplemental analyses provide further support for the conclusion drawn from our primary analysis that self-perceived weight may not independently contribute to long-term decline in metabolic health over-and-above physiological dysregulation from excess adiposity.

**Table S1.** ANCOVA contrasts representing differences in mean self-reported weight comparing metabolically healthy overweight and obese participants who remained metabolically healthy at Wave V with those with became metabolically unhealthy at Wave V

| **Metabolically Healthy Overweight and Obesity Trajectory Group** | **Mean (SD)** |
| --- | --- |
| Group 1: Metabolically healthy with **overweight** at Wave IV 🡪 Metabolically **healthy** at Wave V | 3.56 (0.60) |
| Group 2: Metabolically healthy with **obesity** at Wave IV 🡪 Metabolically **healthy** at Wave V | 4.03 (0.70) |
| Group 3: Metabolically healthy with **overweight** at Wave IV 🡪 Metabolically **unhealthy** at Wave V | 3.52 (0.67) |
| Group 4: Metabolically healthy with **obesity** at Wave IV 🡪 Metabolically **unhealthy** at Wave V | 4.03 (0.78) |
|  |  |
| **Comparison Groups** | **Contrast (95% CI)** |
| Group 1 – Group 2 | **-0.47 (-0.62, -0.31)** |
| Group 1 – Group 3 | 0.042 (-0.13, 0.22) |
| Group 1 – Group 4 | **-0.47 (-0.66, -0.28)** |
| Group 2 – Group 3 | **0.51 (0.33, 0.69)** |
| Group 2 – Group 4 | -0.00080 (-0.17, 0.17) |
| Group 3 – Group 4 | **-0.51 (-0.70, -0.31)** |

Note: **Bold** indicates statistical significance at p < 0.05 level
